# Comparison of brain activity between motor imagery and mental rotation of the hand tasks: a functional magnetic resonance imaging study

**DOI:** 10.1007/s11682-017-9821-9

**Published:** 2018-01-25

**Authors:** Hiroyuki Hamada, Daisuke Matsuzawa, Chihiro Sutoh, Yoshiyuki Hirano, Sudesna Chakraborty, Hiroshi Ito, Hiroshi Tsuji, Takayuki Obata, Eiji Shimizu

**Affiliations:** 1 Department of Cognitive Behavioral Physiology, Graduate School of Medicine, Chiba University, 1-8-1 Inohana, Chuo, Chiba, 260-8670 Japan; 2 Department of Molecular Imaging and Theranostics, National Institute of Radiological Sciences, QST, 4-9-1 Anagawa, Inage, Chiba, 263-8555 Japan; 3 Research Center for Child Mental Development, Chiba University, 1-8-1 Inohana, Chuo, Chiba, 260-8670 Japan; 4 National Institute of Radiological Sciences, QST, 4-9-1 Anagawa, Inage, Chiba, 263-8555 Japan; 5 Research Center for Charged Particle Therapy, National Institute of Radiological Sciences, Chiba, Japan

## Abstract

Motor imagery (MI) has been considered effective in learning and practicing movements in many fields. However, when evaluating the effectiveness of this technique, the examiner has no way of assessing the participant’s motor imagery process. As an alternative, we have been exploring a mental body-part rotation task, in which the examiner can estimate the participant’s motivation and ability to sustain attention through the scored results. In this study, we aimed to investigate the possible application of a mental rotation (MRot) task and used fMRI to compare the brain activity during the MRot task with that during an MI task in healthy volunteers. Increased blood oxygenation level-dependent signals were observed bilaterally in the premotor areas and supplementary motor area during performance of both MI and MRot tasks. Our findings suggest that MRot could be an alternative to MI.

## Introduction

In recent years, motor imagery (MI) tasks have been used in learning and practicing movements in many fields such as sports, dance, music and rehabilitation (Schack et al. 2014; Schuster et al. 2011; Zimmermann-Schlatter et al. 2008). Motor imagery can be defined as “a dynamic state during which an individual mentally simulates a given action” (Decety et al. 1991).).

Mental rotation is performed in the absence of real movements by imagining visual stimuli rotating to an orientation other than that in which they are presented (Shepard and Metzler 1971). When mental rotation concerns a body part, participants tend to imagine the movement of their corresponding body part. Mental rotation of body parts engages anatomically interconnected brain systems that are implicated in the integration of sensorimotor information (Parsons 1987, 1994; Parsons and Fox 1998). In terms of the participants’ orientation toward a task, it generally helps when they are motivated by scored feedback, as if they were playing a game; in fact, this may be a special characteristic for promoting motor learning (Wulf et al. 2010). The MI task depends completely on the participant’s internal motor imagery process, which the examiner has no way of assessing. However, in the mental rotation task, the examiner can at least partly estimate the participant’s motivation and ability to sustain attention through the accuracy of the performance. It should, however, be noted that with regard to the involvement of body parts, the mental rotation task could be affected by the individual’s physical mobility, as in MI. Previous studies reported that the responses were delayed and their accuracy was compromised when the picture stimulus showed the diseased side, e.g., in upper limb or leg amputees or in patients with complex regional pain syndrome (CRPS type 1) accompanied by movement disorders (Nico et al. 2004; Moseley 2004). In a behavioral study of healthy participants, it has also been reported that changes in body position affected the performance of the mental rotation of body parts. For example, in mental rotation tasks involving the hand, reaction time was delayed when the hand was behind the back (Ionta et al. 2009).

Recent neuroimaging research has focused on the functional neuroanatomy of MI and hand mental rotation. Hanakawa et al. (2008) compared the brain activation between actual motion and MI using the tapping of fingers. The distributed activation in motor-related areas was largely the same for the MI task and the actual execution of the movement. Studies on mental rotation tasks involving body parts have shown activation of the movement-related region (premotor area and supplementary motor area) (Kosslyn et al. 1998; de Lange et al. 2006; Zapparoli et al. 2014; Perruchoud et al. 2016), and these domains overlap with those revealed by neuroimaging research on MI (Vingerhoets et al. 2002; Wraga et al. 2003; Zacks 2008; Hetu et al. 2013), which suggests that in performing mental rotation, subjects at least partly use the same strategy as when performing MI. Direct comparisons between the two types of tasks from the very same images can be obtained only from a behavioral study measuring differences in reaction times (Parsons 1994), but such neuroimaging studies have not been performed.

In this study, we evaluated the similarities and the differences between an MI task and a hand mental rotation task from the viewpoint of brain activity observed by fMRI with the same visual images in the same individuals. We believe the results will contribute to evaluations of the potential clinical validity of hand mental rotation as an alternative method to MI.

## Methods

### Participants

The participants for the experiment were 26 healthy right-handed individuals (age 24.6 ± 4.7 years; Edinburgh Inventory score > 80; 13 males). The protocol was approved by the local ethics committee (Chiba University Graduate School of Medicine and Research Center for Charged Particle Therapy, National Institute of Radiological Sciences), and the research was conducted in accordance with the 1964 Declaration of Helsinki. The trial was registered as UMIN ID R000007500. Informed consent was obtained from all study participants.

### Experimental tasks

**Fig. 1:**
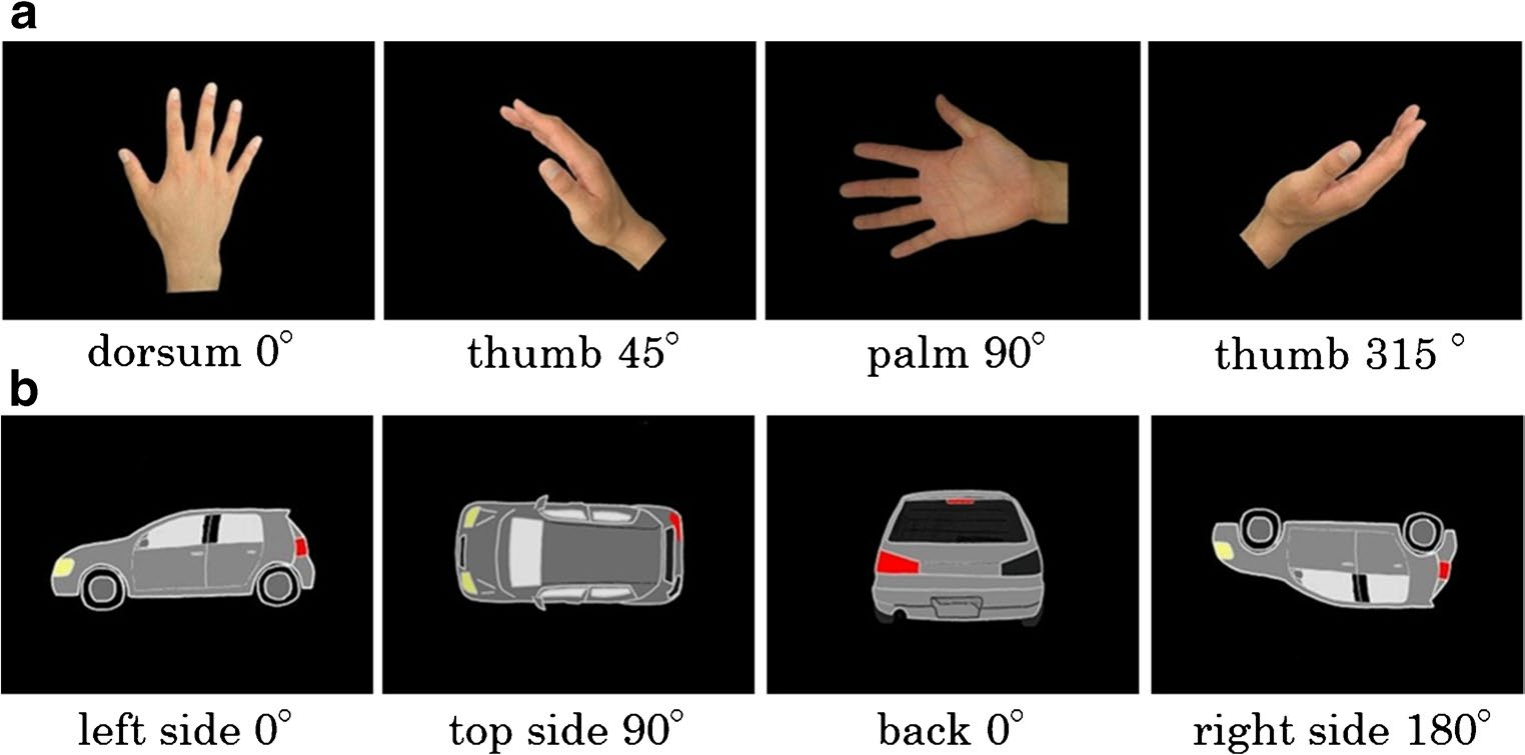
Patterns of stimuli. **a** Motor imagery (MI) task and mental rotation of a hand (H-MRot) task stimuli were presented in three views (dorsum, palm, and thumb) and at four orientations (0, 45, 90, and 315°; the right hand corresponded to counterclockwise rotation, and the left hand corresponded to a clockwise rotation). **b** Mental rotation of an object (O-MRot) task stimuli were presented in three positions and at four angles of orientation (0, 90, 180, and 270°, counterclockwise rotation)

In addition to comparing an MI task and mental rotation of hand task (H-MRot), we added an MRot task involving an object (O-MRot task) to investigate whether brain activity in the mental rotation task differs depending on what the subjects rotate in their mind. Thus, each participant took part in three experimental conditions, namely, the MI task and two mental rotation tasks (object and hand). The MI task stimuli also consisted of photos of a hand. Left and right hands were both pictured; they were mirror images of each other and could be presented in three views (dorsum, palm, and thumb). All stimuli were presented at four orientations (0, 45, 90 and 315°; the right hand corresponded to a counterclockwise rotation, and the left hand corresponded to clockwise rotation) (Fig. 1a). In the trial, the participants had to imagine the movement (kinematic image) they would have to undergo for their own hand to match that in the presented picture. The participants were instructed as follows: “Please imagine moving your hand to fit the presented hand pictures. Please keep your hands unmoved during the task.”

The stimuli for the mental rotation of a hand (H-MRot) task included photos of a hand. In the trial, the participants had to judge whether the hand was a right or left hand. The picture views and orientations used the same patterns as in the MI task.

The stimuli for the mental rotation of an object (O-MRot) task presented a gray car on a computer screen oriented in three positions (back, side and top) and at four angles of orientation (0, 90, 180, and 270°, counterclockwise rotation) (Fig. 1b). The left or right rear light of the car was illuminated red. In the trial, participants had to judge whether the red light was at the right or left rear location. Left and right referred to the perspective from the back of the car; thus, mental rotation of the object had to be performed to answer correctly.

Participants made right/left judgments (H-Mrot and O-Mrot) by pressing an fMRI-compatible button with their right hand (using the middle finger for a right-handed stimulus and the index finger for a left-handed stimulus). The participants were instructed to lie as still as possible to prevent motion artifacts. They were asked to respond as quickly and accurately as possible as to whether the stimulus was a right hand or left hand. Before measurement was begun, each task was practiced to familiarize the subjects with the protocol and confirm their ability to perform it.

While in the scanner, the participants lay supine with the head supported and with foam padding to reduce motion in the volume coil. The task stimuli were projected onto a screen placed at the end of the scanner table, and the participants viewed the screen using an angled mirror mounted above their heads.

### Experimental protocol

**Fig. 2:**
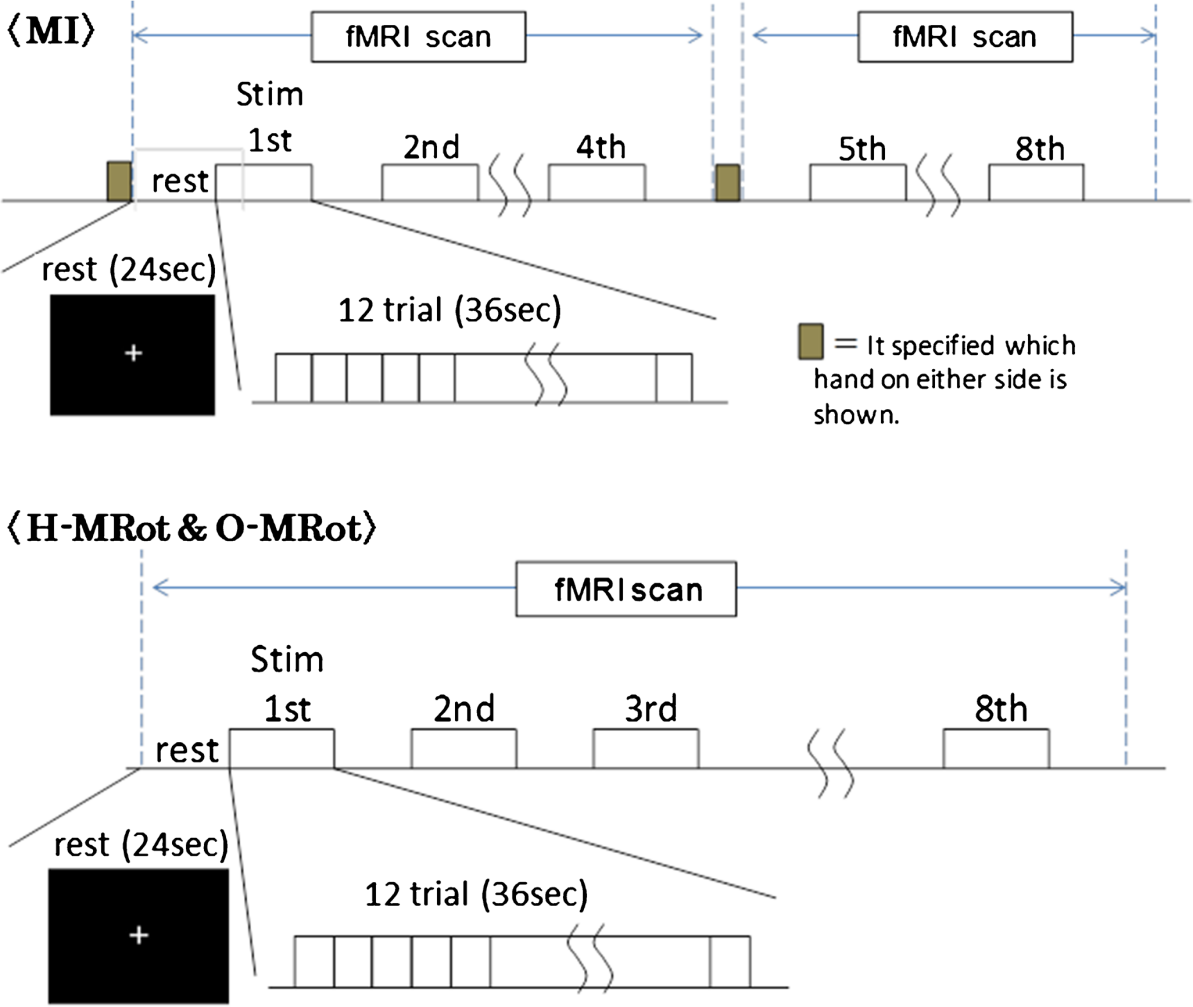
Pattern diagram of tasks. The stimuli were presented in blocks for scanning. Each condition was divided into eight blocks, with the blocks each consisting of 12 stimuli. The timing was stimulus-paced; i.e., after 3 s, a new stimulus appeared regardless of whether the participant had completed the task to control the visual load between conditions. In the motor imagery (MI) task, the stimuli were separated into 4 anterior blocks and 4 posterior blocks (e.g., if right-handed pictures were used in the anterior blocks, left-handed pictures were used in the posterior blocks). The MRot trials were set up so that the stimulus would not be presented to the same side four times or more in a row; otherwise, the pictures were presented at random

The experiment was programmed using E-Prime software (Psychology Software Tools Inc., Pittsburgh, PA) running on a PC. Each condition was divided into eight blocks, with the blocks each consisting of 12 stimuli (total 96 stimuli in each condition) (Fig. 2). The timing was stimulus-paced; i.e., after 3 s (a stimulus was 2700 milliseconds, and a fixation cross was presented for 300 milliseconds after each stimulus), a new stimulus appeared regardless of whether the participant had completed the task to control the visual load between conditions. In the MI task, the stimuli were separated into 4 anterior blocks and 4 posterior blocks (e.g., if right-hand pictures were used in the anterior blocks, left-hand pictures were used in the posterior blocks). In all conditions, the rest period was 24 s with a fixation cross appearing before the first block of stimuli, between each block of stimuli and after the final block of stimuli. The participants were requested to look at the fixation cross. The side that was presented first was determined at random, and the pictures were presented at random. The MRot trials were set up so that the stimulus would not be presented to the same side four times or more in a row; otherwise, the pictures were presented at random.

The three tasks were ordered so that the H-MRot task was performed before the MI task, because the MI task could affect the cognitive style of the H-MRot task. The following three patterns were used: (1) H-MRot / O-MRot / MI; (2) O-MRot / H-MRot / MI; and (3) H-MRot / MI / O-MRot. The patterns were distributed among the participants at random.

### fMRI paradigm

Images were acquired using a 3-T scanner (MAGNETOM Verio 3T, Siemens, Germany). T2*-weighted functional images were acquired using a gradient-echo, echo-planar imaging (EPI) pulse sequence (TR = 3000 ms, TE = 30 ms, flip angle = 75°, matrix 64 × 64, FOV = 240 mm). Thirty-five contiguous 3.8-mm transverse slices were taken. The field of view was aligned parallel with the commissural line and included the full dorsal extent of the brain. A total of 168 whole-brain volumes were acquired during each task. The first 5 volumes were removed from the analysis. High-resolution T1-weighted structural MRI images were also recorded for each participant (TR = 2300 ms, TE = 2.46 ms, matrix 256 × 256, 1-mm slice thickness, FOV = 250 mm).

### Statistical analysis

Data processing was performed with SPM8 (Wellcome Department of Imaging Neuroscience, London, UK). All volumes were realigned to the first reading of each scanning session to correct for subject motion and were spatially normalized into the standard space defined by the MNI template. Further, subject motion was confirmed within 2 mm in each direction. Before statistical analysis was performed, the images were smoothed with an 8-mm isotropic Gaussian kernel to increase the signal-to-noise ratio by attenuating high-frequency noise and to compensate for inter-subject gyral variability. Statistical parametric maps (SPM) were generated on a voxel-by-voxel basis using a general linear model with a hemodynamic model of the three conditions in the experiment. The pre-processed data were set in first-level individual analysis for comparing brain activation during each task with brain activation during the resting period. The t-statistic created subject-specific SPMs. The contrasts were applied to the parameter estimates to determine brain regions showing significant increases in task-related activation associated with each task vs the resting baseline. Additional regressors for subject motion were not used in this experiment because the motions were controlled to be within 2 mm in the experiment.

For group analysis, functional imaging data were collapsed to achieve one representative volume per condition per subject. These condition images were entered into a second-level statistical analysis, thereby affecting a random effects model. SPMs were computed to compare the stimuli vs. the resting condition. Increased blood oxygenation level-dependent (BOLD) signals were calculated and considered to be significant for p < 0.05 (FWE-corrected). Comparisons among the three conditions were analyzed at p < 0.05 (FWE-corrected) and at p < 0.001 (uncorrected) with an extended cluster threshold of 15 contiguous voxels. Coordinates in SPM were adjusted to Talairach and Tournoux atlas brain coordinates using an anatomical chart (Talairach and Tournoux 1988) and the Talairach daemon client (http://www.talairach.org/).

The response times of the MRot tasks were analyzed by paired t-test in relation to laterality (left and right) and repeated-measures analysis of variance (ANOVA), orientation (4 angles) and view (H-MRot tasks; and palm and thumb, O-MRot tasks; three positions). Post hoc comparisons were performed using a Bonferroni correction. The accuracies of the MRot tasks were analyzed by Wilcoxon signed-rank tests, laterality (left and right) and repeated-measures analysis of variance (ANOVA), orientation (4 angles) and view (H-MRot tasks; and palm and thumb, O-MRot tasks; back, side and top). Post hoc comparisons were performed using a Wilcoxon signed-rank test with a Bonferroni correction.

## Results

**Fig. 3:**
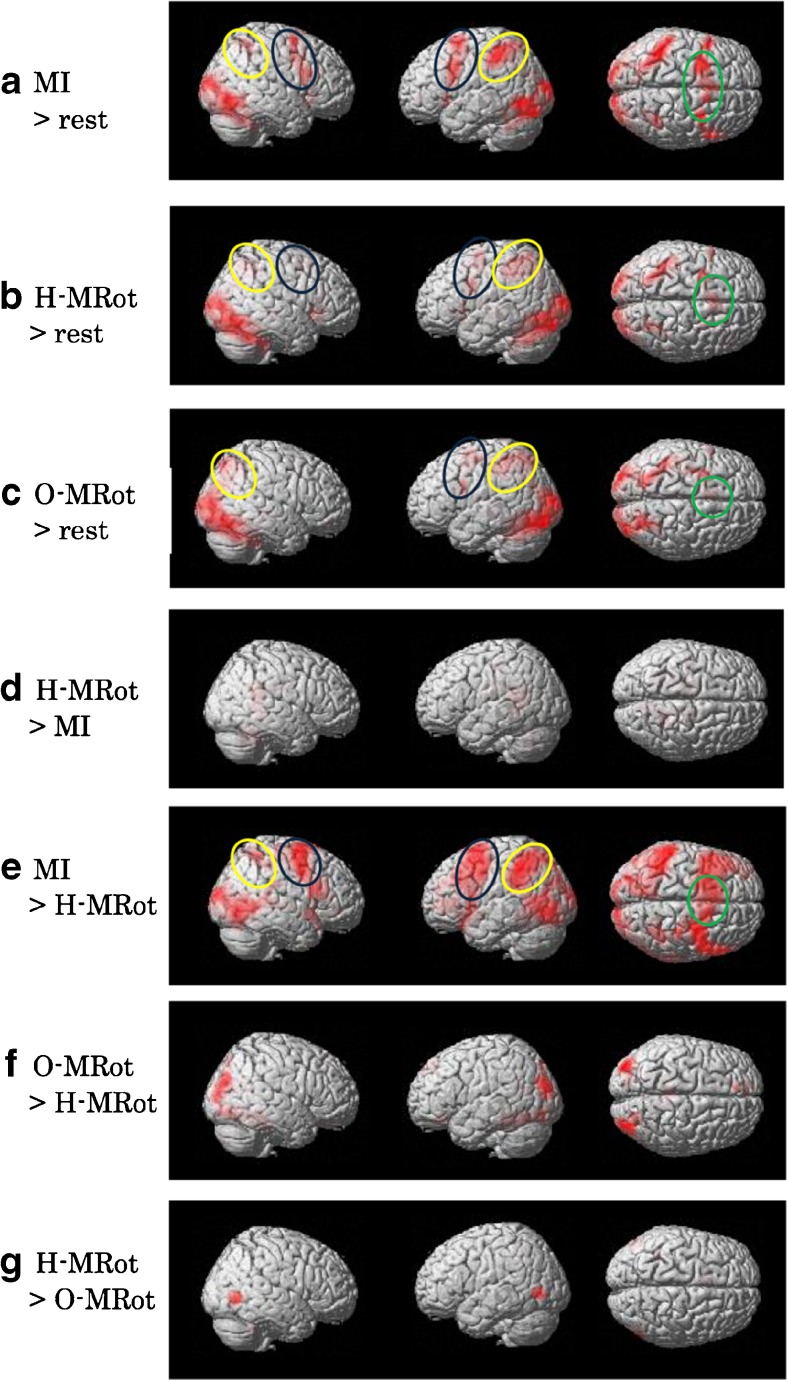
Brain activities in all contrasts. **a** The motor imagery (MI) task evoked activation in the bilateral middle occipital gyrus (BA 37, 18), the bilateral premotor cortex (BA 6), the right inferior parietal lobules (BA 40), and the left superior and inferior parietal lobules (BA7, 40). **b** The mental rotation of a hand (H-MRot) task evoked activation in the bilateral middle occipital gyrus (BA 18), the bilateral premotor cortex and supplementary motor area (BA 6), the right inferior parietal lobules (BA 40), and the left superior and inferior parietal lobules (BA7, 40). **c** The mental rotation of an object (O-MRot) task evoked activation in the bilateral middle occipital gyrus (BA 18) and the right premotor cortex (BA 6). **d-e** Contrasts were defined as follows: H-MRot minus MI tasks and MI minus H-MRot tasks. The premotor area, the supplementary motor area (BA 6), and the inferior parietal lobe (BA 40) were dominant in the MI task. Blue circles indicate activated premotor areas. Green circles indicate activated supplementary motor areas. Yellow circles indicate activated parietal association areas. **f-g** Contrasts were defined as follows: O-MRot minus H-MRot tasks and H-MRot minus O-MRot tasks. Premotor areas were dominant in H-MRot tasks. **a-c**: group analysis, n = 26, P < 0.05, FWE. **d-g**: group analysis, n = 26, P < 0.001, uncorrected

The MI task evoked activation in the bilateral middle occipital gyrus (BA 37, 18), the bilateral premotor cortex (BA 6), the right inferior parietal lobules (BA 40), and the left superior and inferior parietal lobules (BA7, 40) (Table 1; Fig. 3a). The H-MRot task evoked activation in the bilateral middle occipital gyrus (BA 18), the bilateral premotor cortex and supplementary motor area (BA 6), the right inferior parietal lobules (BA 40), and the left superior and inferior parietal lobules (BA7, 40) (Table 1; Fig. 3b). The O-MRot task evoked activation in the bilateral middle occipital gyrus (BA 18), the bilateral superior and inferior lobule (BA 7, 40), and the right premotor cortex (BA 6) (Table 1; Fig. 3c).

**Table 1:** Foci of significant activation and their stereotaxic coordinates for all contrasts

						Coordinates		
Contrast			BA	Cluster	Z score	x	y	z
MI > rest	L	Inferior Temporal Gyrus	37	12,980	7.12	-44	-68	-2
	L	Inferior Parietal Lobule	40		7.08	-36	-41	43
	R	Lingual Gyrus	18		6.91	8	-82	-6
	R	Inferior Frontal Gyrus	47		5.41	34	23	-5
	L	Medial Frontal Gyrus	32	4823	6.76	-6	10	47
	L	Precentral Gyrus	9		6.75	-42	6	33
	L	Sub-Gyral	6		6.56	-28	1	52
	R	Medial Frontal Gyrus	6		5.69	30	5	53
	L	Inferior Frontal Gyrus	47	199	5.54	-38	17	-4
	R	Superior & Inferior Parietal Lobule	7, 40	486	5.50	38	-48	58
H-MRot > rest	R	Inferior Occipital Gyrus	18	23,735	> 7.55	32	-50	-21
	L	Fusiform Gyrus	37		7.54	-42	-59	-7
	L	Inferior Parietal Lobule	40		7.47	-32	-44	43
	R	Superior & Inferior Parietal Lobule	7, 40		6.36	38	-48	58
	L	Inferior Frontal Gyrus	47		5.66	-36	17	-3
	R	Inferior Frontal Gyrus	47	1722	6.01	32	25	-1
	L	Medial Frontal Gyrus & Middle Frontal Gyrus	6, 32	1403	6.96	-6	10	46
	R	Superior Frontal Gyrus	6		5.80	12	12	51
	L	Inferior Frontal Gyrus	9	227	6.58	-59	9	29
	R	Middle Frontal Gyrus	6	182	6.35	26	2	48
	R	Middle Frontal Gyrus	46	55	5.36	46	32	17
	L	Precentral Gyrus	6		4.88	-32	1	24
O-MRot > rest	L	Middle Occipital Gyrus	18	22,792	> 7.62	-28	-91	12
	R	Precuneus & Superior Parietal Lobule	7		7.01	30	-66	35
	L	Inferior Parietal Lobule	40		6.72	-44	-36	50
	L	Precuneus & Superior Parietal Lobule	7		6.65	-22	-60	47
	L	Inferior Frontal Gyrus	9	178	5.80	-53	7	25
	R	Superior Frontal Gyrus	11	28	5.61	30	48	-18
	R	Frontal Lobe,Middle Frontal Gyrus	6	61	5.22	24	2	46

Significance level p < 0.05, FWE-corrected; R: right, L: left, BA: Brodmann area, Cluster: cluster size in voxels, x, y, z: coordinates in Talairach space

In the H-MRot task, significantly greater activations than in the MI task were observed in the right cingulate (BA31), left agranular retrolimbic area (BA 30) and the right occipitotemporal area (BA 37), as shown in Fig. 3d and Table 2 (P < 0.05; FWE-corrected), and as observed in the left post-central gyrus (BA 3) and left insula (BA 13), as shown in Table 3 (p < 0.001; uncorrected). Figure 3e shows that the BOLD signal increased during the MI task compared with the H-MRot task (Tables 2 and 3). Activations in the bilateral occipital cortex (BA 18), the left inferior parietal lobule (BA 40), the premotor cortex (BA 6), and the supplementary motor area (BA 6) were stronger in the MI task than in the H-MRot task (P < 0.05; FWE-corrected). Figure 3f shows the O-MRot task minus the H-MRot task, which showed activation in the left fusiform gyrus (BA37), the parahippocampal gyrus (BA 37) and the bilateral occipital gyrus (BA 19) (P < 0.05; FWE-corrected) (Table 2) and in the areas of the left superior parietal lobule (BA 5, 7) (p < 0.001; uncorrected) (Table 3). Figure 3g shows that the BOLD signal increased during the H-MRot task compared with the O-MRot task. The significant activations in the right occipitotemporal area (BA37), the left supplementary motor area (BA 6), the right insula (BA 13) and left inferior parietal lobule (BA 40) were dominant in the H-MRot task (p < 0.001; uncorrected) (Table 3).

**Table 2:** Foci of significant activation and their stereotaxic coordinates for the comparison between contrasts (FWE-corrected)

						Coordinates		
Contrast			BA	Cluster	Z score	x	y	z
H-MRot >-MI	R	Cingulate Gyrus	31	144	6.36	20	-44	21
	L	Posterior Cingulate	30	75	5.70	-20	-48	13
	R	Parahippocampal Gyrus	37	12	5.46	40	-37	-5
MI > H-MRot	R	Lingual Gyrus	18	382	5.79	22	-76	-3
	L	Inferior Parietal Lobule	40	645	5.55	-55	-37	46
	R	Middle Frontal Gyrus	6	253	5.47	36	-2	44
	L	Inferior Temporal Gyrus	37	323	5.44	-44	-70	0
	L	Inferior Frontal Gyrus	9	6.16	5.15	-44	5	31
	L	Middle Frontal Gyrus	6	141	5.08	-28	0	48
	R	Cuneus	18	141	5.06	16	-92	16
	L	Superior Frontal Gyrus	6	19	5.05	-22	15	62
	L	Precentral Gyrus	44	29	4.99	-51	14	10
	R	Inferior Temporal Gyrus, Inferior Occipital Gyrus	19	50	4.96	44	-72	0
	L	Superior Frontal Gyrus	6	15	4.96	-8	10	51
	R	Middle Frontal Gyrus	6	75	4.91	55	6	38
	L	Middle Frontal Gyrus	9	17	4.86	-44	31	33
O-MRot > H-MRot	L	Fusiform Gyrus	37	393	6.16	-30	-47	-11
	R	Parahippocampal Gyrus	37	330	5.68	34	-41	-10
	R	Fusiform Gyrus	20		5.62	30	-36	-17
	R	Superior Occipital Gyrus	19	149	5.64	38	-80	28
	L	Superior Occipital Gyrus	19	135	5.41	-34	-82	23
H-MRot > O-MRot	No significant							

Significance level p < 0.05, FWE-corrected; R: right, L: left, BA: Brodmann area, Cluster: cluster size in voxels, x, y, z: coordinates in Talairach space

**Table 3:** Foci of significant activation and their stereotaxic coordinates for the comparison between contrasts with uncorrected analysis except for the results shown by FWE analysis

						Coordinates		
Contrast			BA	Cluster	Z score	x	y	z
H-MRot >-MI	L	Postcentral Gyrus	3	50	3.55	-42	-19	47
	L	Insula	13	32	3.52	-42	-7	15
MI > H-MRot	R	Middle Frontal Gyrus	47	58	3.75	50	46	-7
O-MRot > H-MRot	R	Fusiform Gyrus	20	3833	5.62	30	-36	-17
	L	Posterior Cingulate	30	551	4.51	-20	-54	17
	L	Precuneus	7	662	4.01	-10	-35	42
	R	Cingulate Gyrus	31		3.67	16	-35	37
	R	Paracentral Lobule	5		3.55	6	-31	49
	R	Middle Frontal Gyrus	11	51	3.83	26	26	-18
	L	Medial Frontal Gyrus	11	177	3.82	-6	26	-15
	L	Medial Frontal Gyrus	10	24	3.52	-6	54	-8
	L	Superior Frontal Gyrus	8	210	3.50	-6	41	42
	L	Inferior Frontal Gyrus	47	52	3.41	-44	30	-17
H-MRot > O-MRot	R	Inferior Temporal Gyrus	37	283	4.42	53	-68	-2
	L	Inferior Temporal Gyrus	37	234	4.16	-50	-70	2
	L	Medial Frontal Gyrus	6, 32	63	3.57	-10	14	45
	R	Insula	13	43	3.35	30	27	2
	L	Inferior Parietal Lobule	40	18	3.25	-36	-42	48

Significance level p < 0.001, uncorrected; R: right, L: left, BA: Brodmann area, Cluster: cluster size in voxels, x, y, z: coordinates in Talairach space

**Fig. 4:**
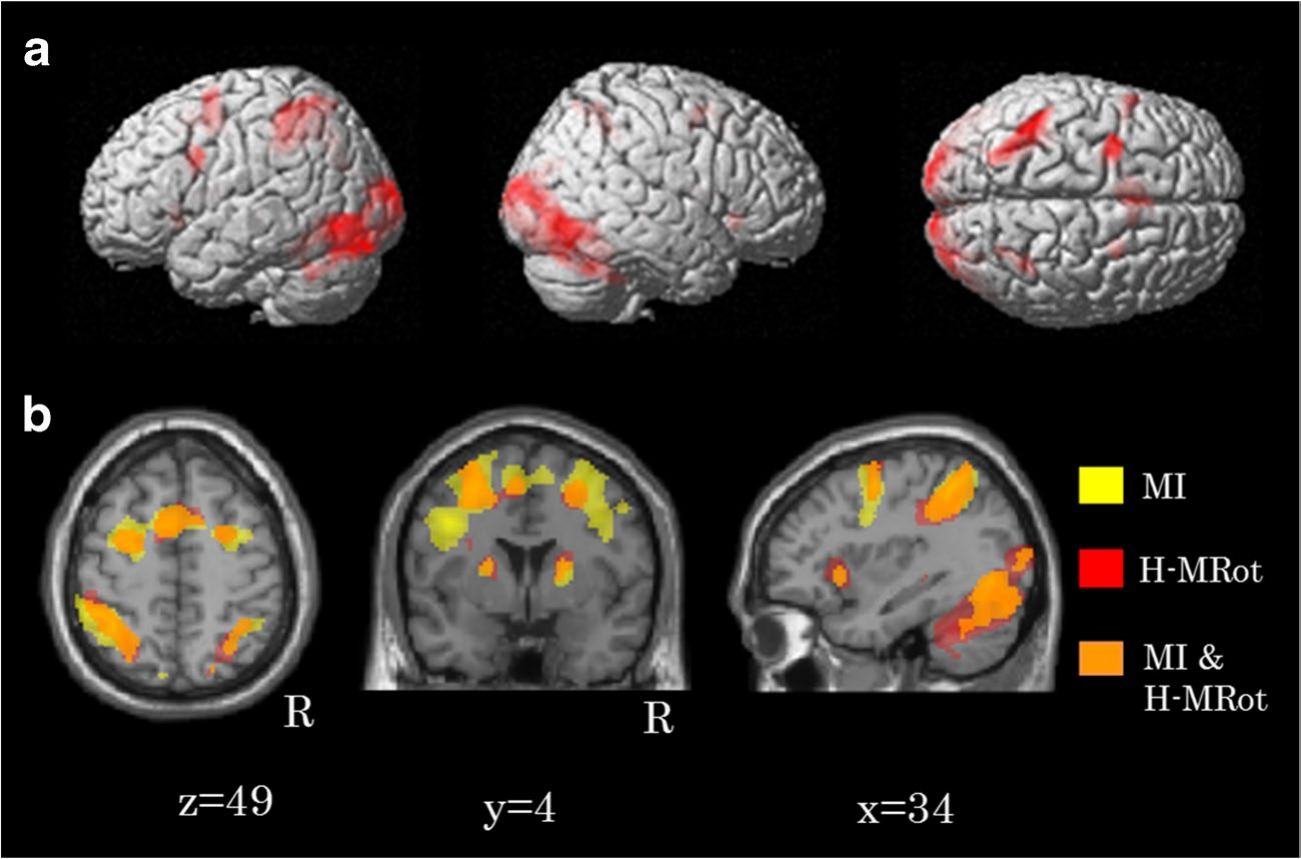
Activation areas in MI and H-MRot tasks. **a** In the motor imagery (MI) task and the mental rotation of a hand (H-MRot) task, common activation areas were observed in the bilateral premotor area (BA 6), the supplementary motor area (BA 6), the right inferior parietal lobules (BA 40), the left parietal association area (BA 7, 40), and the bilateral visual area. **b** Yellow areas indicate the activity areas in the MI task and red areas indicate the activity areas in the H-MRot task. Orange areas are co-activation areas. Group analysis, n = 26, P < 0.05, FWE

Activation areas common to both the MI task and H-MRot task included the bilateral occipitotemporal area (BA37), the bilateral premotor area (BA 6), the supplementary motor area (BA 6) and the left parietal association area (BA 7, 40) (Table 4; Fig. 4). Figure 4b indicates the areas activated separately under the two conditions (yellow, MI task; red, H-MRot task) and the areas of overlap (orange).

**Table 4:** Foci of significant activation and their stereotaxic coordinates for the coactivation areas in MI and H-MRot tasks

					Coordinates		
Contrast			BA	Cluster	x	y	z
MI & H-MRot	L	Inferior Temporal Gyrus	37	11,152	-44	-68	-2
	L	Inferior Parietal Lobule	40		-36	-41	43
	R	Lingual Gyrus	18		8	-82	-6
	L	Medial Frontal Gyrus	32	1068	-6	10	47
	L	Sub-Gyral	6		-28	1	52
	R	Superior Frontal Gyrus	6		10	10	53
	L	Inferior Frontal Gyrus	9	201	-42	5	31
	R	Middle Frontal Gyrus	6	145	30	5	53
	L	Inferior Frontal Gyrus	47	98	-38	17	-4
	R	Superior & Inferior Parietal Lobule	7, 40	321	38	-48	58
	R	Inferior Frontal Gyrus	47	144	34	23	-5

Significance level p < 0.05, FWE-corrected; R: right, L: left, BA: Brodmann area, Cluster: cluster size in voxels, x, y, z: coordinates in Talairach space

In terms of the reaction times of the H-MRot tasks, laterality, orientation and view showed no significant differences (t = 0.475, p > 0.05), (F (3, 100) = 0.476, p > 0.05) (F (2, 75) = 1.953, p > 0.05). In terms of the reaction times of the O-MRot tasks, laterality and view showed no significant differences (t = 1.166, p > 0.05), (F (2, 75) = 2.77, p > 0.05). Only position was a significant factor (F (3, 100) = 14.978, p < 0.01). Post hoc comparison showed that 180° was slower than the other angles (all comparison, p < 0.001) (Table 5).

**Table 5:** Mean response times and mean percent accuracy of H-MRot tasks

	Laterality		Views			Orientations (angle)			
	Right	Left	dorsum	palm	thumb	0	45	90	315
Response times (msec)	1032.00	1023.13	1104.29	974.59	1003.91	992.98	1008.24	1044.79	1064.51
(SE)	47.96	45.22	45.35	50.51	50.01	42.85	53.02	47.04	46.74
Accuracy (%)	93.40	93.96	94.28	94.16	94.88	95.08	94.96	94.56	94.76
(SE)	0.48	0.45	0.43	0.44	0.26	0.23	0.29	0.29	0.32

*SE* standard errors

In terms of the accuracy of the H-MRot tasks, the laterality, orientation and view showed no significant differences. In terms of the accuracy of the O-MRot tasks, laterality did not differ significantly, but differences in orientation were significant in that 180° was worse than 270° (p = 0.009); additionally, the view showed significant differences in that the side view was worse than the other views (compared to back: p < 0.01, top: p = 0.011) (Table 6).

**Table 6:** Mean response times and mean percent accuracy of O-MRot tasks

	Laterality		Views			Orientations (angle)			
	Right	Left	back	side	top	0	90	180	270
Response times (msec)	802.36	784.99	742.31	850.39	788.26	683.72	742.41	993.77	754.74
(SE)	34.49	29.85	33.43	36.78	29.18	34.76	28.06	48.02	26.59
Accuracy (%)	94.16	94.20	95.20	94.16	95.00	95.48	95.16	94.48	95.24
(SE)	0.48	0.41	0.25	0.38	0.34	0.24	0.31	0.28	0.27

*SE* standard errors

## Discussion

Previous studies have reported that the brain activity that occurs during the H-MRot task involves the premotor area, supplementary motor area, parietal association area, and visual area (Kosslyn et al. 1998). Those same areas of activity were also observed in the present study, and the activation was stronger in the left hemisphere, as in the previous study (Kosslyn et al. 1998). It is known that the premotor area of the left hemisphere is activated strongly when healthy, right-handed subjects are judging the choice of joints to move and the sequence of a hand movement (Schluter et al. 2001; Hlustik et al. 2002). However, although a predominance of activation was shown in the left hemisphere, activation of the premotor area in the right hemisphere was also observed. Generally, in the movement of the limbs, the motor area and the premotor area on the opposite side show significant activation (Naito et al. 2002; Wolbers et al. 2003). However, the participants were requested to push buttons with their right index or middle fingers to answer; it was possible that the brain activities of the relevant motor-related area were affected. Although such an effect, if any, is expected to be small because of the simplicity of the movements, it is difficult to draw definite conclusions about the laterality of the brain in this study.

Comparable activation to the MI task would need to be demonstrated for the H-MRot task for the H-MRot task to be proposed as an effective alternative to the MI task. Importantly, common activations of MI and MRot tasks involving body parts were suggested in the previous behavioral testing and neurophysiological experiments (Nico et al. 2004; Ionta and Blanke 2009; Wraga et al. 2003; Vingerhoets et al. 2002; Osuagwu and Vuckovic 2014). In our MI task and H-MRot task, common activations were in fact observed in the bilateral premotor areas, the supplementary motor area, the bilateral left parietal association area, and the visual areas of both sides. Activation of the premotor area and supplementary motor area in the MI task (Ehrsson et al. 2003) might support the theory that the relevant movement was imagined in the H-MRot task (i.e., the subjects implicitly think of the sequence and choice of movement, e.g., how to move the shoulder, elbow, wrist, and forearm). Our results suggested that the H-MRot task might, at least partly, involve the same mental processes used to carry out the MI task. However, the scale of the activation during H-MRot was much smaller than that during the MI task. It is possible that MI promotes greater activation of the motor-related areas, and the strength of an individual’s memory regarding the movement of body parts might be involved. It has been shown that MI tasks help improve performance (Yue and Cole 1992; Guillot et al. 2010), and it is known that the brain activity in the MI is similar to that observed during actual movement (Decety et al. 1991; Jeannerod 2001). Thus, our results and the previous data suggest that when ideally performed, MI tasks might be more useful for the improvement of motor performance than H-MRot tasks from the viewpoint of brain activation. In fact, in a randomized trial for stroke patients lasting six weeks, a mental practice (MI) intervention group showed significant improvement when compared with a control group (Page et al. 2005). In the visual cortex, stronger activation was observed in the MI tasks than in the H-Rot tasks, which might be a reflection of the difference in the durations of the visual presentations in the two tasks. The MI tasks required visual concentration while a picture was being presented (see Table 1, 2700 milliseconds), but the H-MRot tasks required visual concentration only until the subject provided an answer (see Table 1, 1027.57 s in average).

The advantages of H-MRot over MI should be considered. The H-MRot tasks more strongly evoked the brain activities of the posterior cingulate and parahippocampal regions than the MI tasks. The posterior cingulate is one of the regions of the default mode network, which might be responsible for arousal, awareness, internally directed thought, and controlling the attention between internal and external environments (Leech 2014). In comparison with MI tasks, the participants had to pay close enough attention to provide an “answer” in the H-MRot tasks; the demand for arousal might have activated this region. The parahippocampal region has been reported to be involved in visuospatial cognition (Aminoff, 2013). Thus, the H-MRot tasks may have recruited more visual spatial information processing. In addition, the H-MRot task can provide objective feedback. The scoring of their answers would motivate participants, and such feedback has been shown to have special importance in promoting motor learning (Wulf et al. 2010). We expect that the feedback in the H-MRot task could make the participants feel that doing the task is like playing a game, which would engage them in the H-MRot task more readily than in the MI task. As mentioned in the introduction, the MI task totally depends on the participant’s motor imagery process in their own minds, which the examiner has no way of accessing. However, in the H-MRot task, the examiner can at least partly estimate the participant’s motivation and ability to sustain attention through the scored results.

In this study, we added the O-MRot task to investigate whether brain activity in the mental rotation task differs depending on what the subjects rotate in their mind. According to Vingerhoets’ (Vingerhoets et al. 2002) report, activation of the premotor area in both sides was observed in both the H-MRot and O-MRot tasks, while in our study, the laterality of activation (left > right) was observed more intensely in the O-MRot task than in the H-MRot task. In their O-MRot tasks, which involved pictures of tools that are manipulated by the hands (e.g., a pencil sharpener, spoon), the subjects’ brains were activated in the motor-related area of the left hemisphere, most likely because the subjects imagined moving the right (dominant) hand to use the tools pictured. In our study, when subjects were performing the O-MRot tasks, some subjects might have imagined grasping the objects as a toy, as in the previous study (Fiorio et al. 2007). Moreover, our O-MRot tasks also required the subjects to imagine three dimensions. It has been reported that the activity of the premotor area increases as subjects perform mental rotation of object tasks with three-dimensional imaginary rotation (Kawamichi et al. 2007). In addition, the activity of bilateral occipitotemporal cortices was significantly increased in the H-MRot tasks compared with O-MRot tasks. The activity of this area can be explained in that it is known to be evoked specifically when body parts are visually presented (Orlov et al. 2010; Bracci et al. 2015).

Behavioral data showed no significant difference of reaction times and accuracies for H-MRot tasks. Our study used limited angles; there was no significant difference between 0° and 90° as in the previous studies (Parsons 1987; Takeda et al. 2010). However, the tendency for a delay in reaction times with an increasing angle was confirmed. At 315°, the reaction time was slower than that at 45° (but not significantly), which could suggest that the anatomical model is involved (it is more difficult to move and therefore to imagine moving to a 315° angle than to a 45°). In the O-MRot task, a significant difference was observed as in the previous study (Shepard and Metzler 1971). This can be inferred to be purely a delay in rotating the object in the brain.

Some limitations should be considered. First, the experiments required the participants to respond with a certain movement in the MRot tasks, namely, pushing a button; this might have affected the brain activity during the tasks, at least to some extent. Second, some angles of the positions in the pictures were very difficult to imagine, and in such case, the brain activity resulting from this kind of intervention would be reduced. If the nearest movement that the subject can produce deviates too much from the photograph shown, it will be difficult for the intervention to have an effect. Further verification of these findings is necessary. In addition, it is important to note that the MI task cannot improve the performance when the task requires subjects to imagine a movement well beyond their actual performance ability (Mulder et al. 2004). Thus, the tasks should ideally be customized to the subjects according to their disabilities.

## Conclusion

We observed brain activities in three conditions and found that both mental rotation tasks (hand and object) activated the motor-related areas in the same way that motor imagery (MI) tasks did. We found that the hand mental rotation tasks (H-MRot task) showed comparable brain activities with MI tasks in the bilateral premotor areas and supplementary motor areas. Our findings suggest that performing H-MRot tasks could be a useful alternative method with some advantages over MI.
